# Probiotics prevent mortality of thermal-sensitive corals exposed to short-term heat stress

**DOI:** 10.1093/ismeco/ycaf039

**Published:** 2025-03-02

**Authors:** Mareike de Breuyn, Malte Ostendarp, Yusuf C El-Khaled, Neus Garcias-Bonet, Susana Carvalho, Christian Wild, Raquel S Peixoto

**Affiliations:** Marine Ecology Department, Faculty of Biology and Chemistry, University of Bremen, Bremen 28359, Germany; Marine Ecology Department, Faculty of Biology and Chemistry, University of Bremen, Bremen 28359, Germany; Red Sea Research Center, Division of Biological and Environmental Science and Engineering, King Abdullah University of Science and Technology, Thuwal 23955, Saudi Arabia; Red Sea Research Center, Division of Biological and Environmental Science and Engineering, King Abdullah University of Science and Technology, Thuwal 23955, Saudi Arabia; Red Sea Research Center, Division of Biological and Environmental Science and Engineering, King Abdullah University of Science and Technology, Thuwal 23955, Saudi Arabia; Marine Ecology Department, Faculty of Biology and Chemistry, University of Bremen, Bremen 28359, Germany; Red Sea Research Center, Division of Biological and Environmental Science and Engineering, King Abdullah University of Science and Technology, Thuwal 23955, Saudi Arabia

**Keywords:** beneficial microorganisms for corals, microbiome restoration, *Acropora hemprichii*, *Pocillopora verrucosa*, coral holobiont function

## Abstract

The use of coral probiotics, i.e. beneficial microorganisms for corals (BMCs), is a novel approach to enhancing coral health under heat stress. While BMCs mitigate coral bleaching and mortality during prolonged heat stress conditions, their effectiveness in mitigating short-term acute heat stress remains understudied. This study investigates the effects of BMCs on two Red Sea hard coral species, *Acropora* cf. *hemprichii* and *Pocillopora verrucosa*, during short-term heat stress. Twelve coral fragments per species were allocated to each treatment across two temperature regimes (26°C and 32°C) for 48 hours, with half receiving BMC inoculation and half serving as controls. Results show BMC supplementation significantly prevented mortality in *Acropora* cf. *hemprichii* at 32°C, contrasting with a 100% mortality observed in the control group. Specifically, probiotic-inoculated *Acropora* cf. *hemprichii* at 32°C exhibited preserved primary production, a 12–13 fold increase in algal cell densities, 4–5 times higher *F_V_/F_m_* ratios, and 4–5 and 2–3 times higher chlorophyll *a* and *c_2_* concentrations, respectively, compared to their untreated conspecifics. All *P. verrucosa* colonies survived the 32°C exposure without tissue loss or reduced holobiont function in both control and BMC treatments. These findings underscore the rapid effects of BMC inoculation, initiated just 2 hours prior to acute heat stress, in protecting heat-sensitive *Acropora* cf. *hemprichii* against mortality and adverse photo-physiological changes, with beneficial effects visible within 2 days. Recognizing the critical timeframe for beneficial effects is paramount for management strategies to address heat-sensitive corals on natural reefs, such as implementing probiotic interventions before anticipated marine heatwaves.

## Introduction

Coral reefs are critically important due to their role in supporting biodiversity, protecting coastlines from erosion, providing habitats for numerous marine species, and contributing significantly to global economies through tourism and fisheries [1–3]. Yet they are critically endangered by a combination of global and local stressors, such as global warming, pollution, and overfishing, resulting in mass die-offs often due to pathogens [4–10]. Thermal stress events, characterized by elevated sea surface temperatures and intensified by climate phenomena like El Niño, have resulted in short- to long-term acute heat stress affecting corals worldwide. This has culminated in four devastating coral mass bleaching events over the past four decades [11], followed by a widespread loss of coral cover as corals become more susceptible to disease [11–13]. Coral bleaching is a phenomenon where the single-celled Dinoflagellates (Symbiodiniaceae) that live within the corals’ gastrodermal cells are expelled by their hosts [14, 15] or photosynthetic pigments of Symbiodiniaceae are lost [16] when a thermal threshold is reached. Coral hosts are dependent on their endosymbiotic algal partner (Symbiodiniaceae) for up to 90% of their nutritional demand depending on the coral species, making symbiosis crucial for coral survival and health [17]. As marine heat waves become more intense and frequent and considering the anticipated profound environmental changes in the future [18], it is urgent to investigate nature-based solutions that mitigate thermally induced coral bleaching and mortality, aiding ongoing reef conservation efforts.

Advancements in coral microbiology have shifted attention from the coral host’s symbiotic relationship with Symbiodiniaceae to a more pronounced dependence on other microorganisms closely associated with corals, particularly bacteria [19–25]. This shift established a foundation for active interventions, such as the use of coral probiotics, i.e. beneficial microorganisms for corals (BMCs; [26]) - a promising strategy to enhance coral tolerance to thermal stress through microbiome restoration and rehabilitation [26, 27]. BMCs are based on native coral-associated bacteria that are selected, assembled to aggregate several putative beneficial mechanisms and re-applied to corals. The selection of bacterial strains for BMC treatments is based on phenotypic traits, such as the reduction of toxic compounds and provision of nutrients, that can improve coral fitness and increase coral resilience and survival [26–30]. Under stress conditions, beneficial microbes naturally found in corals can be replaced by pathogens [31]. The restoration of the coral microbiome can ensure the natural balance within the holobiont*,* by actively mitigating stress and impacts of toxic compounds, hampering pathogen overgrowing and facilitating microbiome restructuring associated with coral health [27].

Previous *ex situ* studies with Scleractinia have demonstrated the substantial potential of BMCs in mitigating coral bleaching induced by thermal stress [30, 32–34], reducing coral mortality [35] and minimizing thermal stress and pathogen driven impacts on the coral [36, 37]. BMCs have further been applied to promote coral growth [38], and alternative microbial therapies have also demonstrated their potential to enhance bleaching resistance [39]. [40] further demonstrated that in situ inoculation of BMCs resulted in shifts of the microbial community without affecting the taxonomic composition and diversity of the microbiome in the surrounding seawater and sediments. These combined findings highlight the promising potential of utilizing BMCs on natural coral reefs and indicate a viable pathway for mitigating coral bleaching and mortality in the face of rising sea temperatures.

Although the application of BMCs holds promise, our understanding of their beneficial effects on the coral holobiont function is still in its infancy. While positive effects of BMCs on coral holobiont function were previously shown to occur over mid-term acute heat stress of 26, 34, and 39 days [30, 32, 34], and long-term acute heat stress of 75 days [35], the current study successfully fills a gap in knowledge by demonstrating that positive effects of BMCs can be observed within short-term acute heat stress (two-day period). In this study, we tested the capacity of BMC inoculation in two Red Sea Scleractinia to mitigate heat stress during a 2-day heat stress laboratory experiment. Our results showed the ability of BMCs to prevent mortality, maintain primary production processes similar to non-stress conditions, and positively impact the photo-physiology of heat-stressed *Acropora* cf. *hemprichii* after 2 days. Contrasting, the coral holobiont of *P. verrucosa* showed no signs of heat stress during the experiments and, therefore, no discernible effect of BMC treatment on holobiont health.

Our findings underscore the importance of accounting for species-specific responses to thermal stress and their potential impact on the observed outcomes of probiotic treatments in corals. The 2-day heat stress period was chosen to better understand the onset of BMC-induced changes on coral holobiont function, allowing for the observation of early beneficial effects on coral photo-physiology. Recognizing the critical timeframe for achieving beneficial effects is essential for developing effective strategies to support heat-stressed corals on natural reefs, including the timely implementation of probiotic interventions before anticipated marine heatwaves.

## Materials & methods

### Experimental design

Study organisms were collected at the coral probiotics village (22°18′307.1”N 38°57′8741″E), a natural coral reef in the Red Sea, Saudi Arabia, designated as an experimental research site [41]. We selected the scleractinian corals *A. hemprichii* and *P. verrucosa* for our experiments based on their distinct responses to environmental stress, as observed in a study by [42], with *A. hemprichii* demonstrating a more flexible microbiome and *P. verrucosa* maintaining a comparatively stable microbial community. The coral species were identified morphologically in the field based on established visual characteristics. We marked 12 coral colonies (six per species) at the coral probiotics village with buoys to allow for repeated sampling of 4–7 cm sized fragments throughout the experiment.

*A. hemprichii* and *P. verrucosa* are reef-building corals that are found in the Red Sea and the Western Indian Ocean [43] and are commonly observed in the coastal waters of Jeddah region, Saudi Arabia. Both *A. hemprichii* and *P. verrucosa* are listed as endangered on the IUCN Red List of Threatened Species [44, 45]. Sailing licenses to the coral probiotics village were issued by the Saudi Coastguard Authority under the auspice of King Abdullah University of Science and Technology (KAUST), which included permission to collect corals for research purposes (IBEC protocol number 22IBEC003_v4).

Collected coral fragments of *Acropora* cf. *hemprichii* and *P. verrucosa* were kept in holder tanks with natural seawater for 1–2 hour (maintained at 26°C and 39.5 PSU). Subsequently, the coral fragments were randomly distributed into two separate 30 L tanks, with one tank assigned to each coral species. Each species received treatment with its specifically formulated BMC consortia. Both tanks were equipped with an IP68 heater and a PULACO 50GPH aquarium pump to ensure a constant temperature of either 26°C or 32°C and continuous water flow. Each heater was connected to a separate SCHEGO TRD112 thermostat and TRD temperature controller to allow for precise control of the water temperature. Above each tank, we installed a lamp setup to maintain light levels close to conditions on the natural coral reef on a day-night rhythm of 12:12 hour. We tested water variables in the mornings and evenings during each treatment using a YSI605596 handheld multiparameter meter and adjusted the temperature and salinity to achieve similar conditions in both tanks. Natural seawater with salinity from 39.06 PSU to 39.93 PSU was used for the experiment and directly collected on the natural reef together with the coral fragments on the day of each treatment.

We used two control treatments with no BMC addition at 26 and 32°C (CT26 and CT32, respectively), and two BMC inoculation treatments at 26 and 32°C (BMC26 and BMC32, respectively) with the application of BMC I and BMC II, and allocated 12 fragments per species to each treatment. BMC I is the tailored treatment for *P. verrucosa* and contains six bacterial strains (*Halomonas piezotolerans, Bacillus aequororis,* two *Pseudoalteromonas* spp*.,* and two *Cobetia* spp*.*), while BMC II is the tailored treatment for *A. hemprichii* and contains four bacterial strains (*Halomonas piezotolerans*, *Cobetia* spp., *Pseudoalteromonas* spp., *Pseudoalteromonas lipolytica*). The bacteria for BMCs were taken from the biobank at the Red Sea Research Center and specially cultured every week. To achieve the tailored combination of microbes in the BMCs, bacterial strains have been isolated from coral mucus and tissue samples of *Styllophora pistillata, Galaxea fascicularis, P. verrucosa*, and *A. hemprichii* and have been screened for beneficial traits in previous research efforts [26].

**Figure 1:**
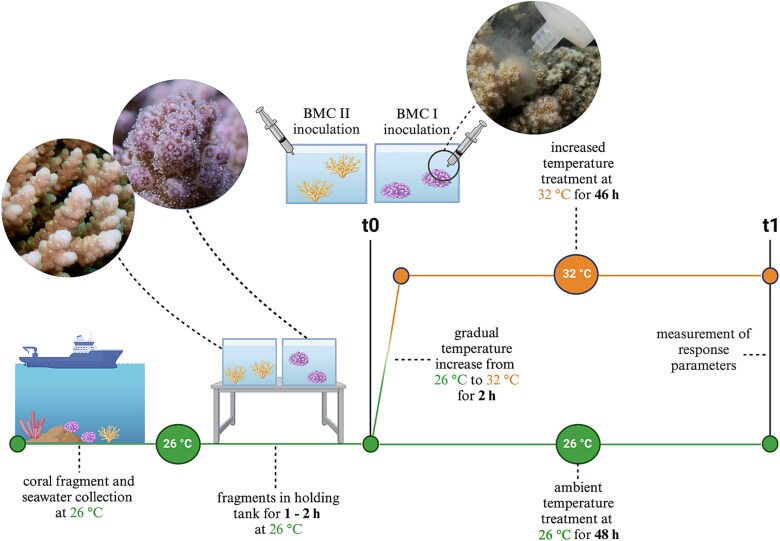
Schematic representation of the experimental design. The following treatments were conducted: BMC26 (n = 6), BMC32 (n = 6), CT 32 (n = 6), CT26 (n = 6). For each treatment, *P. verrucosa* (depicted in the middle image) and *Acropora* cf. *hemprichii* (depicted in the left image) were placed in two separate randomly distributed tanks. At t0, BMC I (*P. verrucosa*) or BMC II (*Acropora* cf. *hemprichii)* were added at two temperature regimes: Ambient temperature maintained at 26°C (BMC26) or increased temperature, gradually increased from 26°C to 32°C over 2 h, and then maintained at 32°C (BMC32). As a control, a parallel setup of the same experiment was carried out at both temperature regimes where fragments received no BMC addition (CT26, CT32).

We added BMCs to the treatments BMC26 and BMC32 at t0 (Fig. 1) using syringes filled with 30 ml of either BMC I or BMC II cells suspended in a saline solution at 10^7^ cells/ml, which was evenly distributed onto each coral fragment (2.5 ml per fragment). This resulted in a final BMC concentration of 10^4^ cells/ml in a tank volume of 30 L. The coral fragments were kept in the BMC-inoculated tank water for 48 hour. To achieve the increased temperature treatments CT32 and BMC32, we raised the temperature from 26 to 32°C in the initial 2 hour of the 48 hour treatment. As a result, the coral fragments were subjected to acute heat stress for 46 hour. The central Red Sea experiences summer temperatures ranging from 30°C to 33°C, making 32°C a representative elevated temperature often encountered by corals on these reefs [46]. The rapid temperature increase facilitated the assessment of probiotic efficacy under extreme conditions, similar to methods used in other studies to evaluate Red Sea coral responses to short-term heat exposure [47]. Conducting experiments during winter ensured stable environmental conditions at the time frame of the fragment collections, minimizing confounding variables and allowing the observed effects to be primarily associated with the induced thermal stress during the ex-situ experiment. We refrained from conducting any water changes due to the short duration of the experiment. After completion, 12 fragments (one per colony of each species) were frozen at −20°C for later analysis (Symbiodiniaceae cell density and chlorophyll concentrations). The remaining 12 fragments (one per colony of each species) were photographed to assess survival, then placed in a controlled setup for primary production measurements, followed by assessments of photosynthetic efficiency. This resulted in 24 coral fragments per treatment, with an n-value of six per species, and a total of 96 coral fragments for the experiment. Experiments were conducted back-to-back to avoid significant environmental background changes (CT26 on 09/02/23, BMC26 on 21/02/23, CT32 on 26/02/23, BMC32 on 02/03/23) with a total of 21 days between the first and the last treatment.

### Coral host analyses

#### Survival by visual assessment

Host survival was assessed by identifying coral fragments as “living” or “dead” after each 48-hour treatment period. A coral was defined as “living” when it had >75% of the total surface area (SA) covered with intact tissue and < 25% SA with tissue necrosis. A coral was defined as “dead” when it had >75% SA covered with tissue necrosis. Necrotic tissue was characterized as pale and/or disintegrating, while intact tissue was characterized as coloured, dense, and firm. For this, we modified the approach outlined by [48], where the percentage of tissue loss was utilized as a metric for analysing coral mortality. We created 3D models of all fragments using Autodesk ReCap Pro version 23.0.0.216 at the beginning of the experiment (t0) and after 48 hours (t1) and visualized potential changes in survival with Adobe Illustrator version 27.6.

#### Primary production by oxygen (O_2_) flux incubations

To establish oxygen consumption rates in the dark (dark respiration, R), and oxygen production in the light (net photosynthesis, P_net_) and to understand how they differ between treatments, we adopted the methodology outlined by [49] for the soft coral *Xenia umbellata*. We conducted O_2_ flux measurements for six coral fragments per species for each treatment and three control blank incubations per species for each treatment, resulting in 18 incubations per treatment. Control blank incubations were conducted to measure planktonic background metabolism with treatment seawater taken from the respective coral tanks (three with seawater from *P. verrucosa* tank and three with seawater from *Acropora* cf. *hemprichii* tank). To assess R and P_net_ rates, coral fragments were directly transferred to incubation chambers (WECK Rundrandglas 100) at the end of the 48 hour treatment period. The chambers had a volume of 1 L and were filled with natural seawater at 26°C or 32°C according to the respective treatment. The seawater for the chambers was collected at the coral probiotics village on the previous day and stored in a temperature-controlled incubator at 26°C. We sealed the chambers gas-tight, carefully avoiding any air bubbles inside the chamber, and placed them into a temperature bath for 1 hour in the light and 1.5 hour in the dark. Each incubation chamber was equipped with a magnetic stirrer (2.5 cm x 0.8 cm) and placed on a stirring plate (HANNA instruments; magnetic stirrer HI 200 M; Speed 60/100) to provide water mixing and to make sure the oxygen concentrations throughout the water column of the chambers were homogenous. We measured O_2_ concentrations using a Multi 3500i/SET optode sensor before and after each incubation, resulting in two measurements for light and dark respectively. To calculate P_net_ in the light and R in the dark, we subtracted start from end concentrations and normalized the result by incubation duration (h). We further standardized control blank O_2_ fluxes by incubation duration (h) and then subtracted the results from the coral incubations. We calculated P_net_ in the light and R in the dark using equation 1. To compute SA, we modelled each fragment and calculated SA using Autodesk ReCap Pro version 23.0.0.216.


(1) \begin{equation*} {P}_{net\ (light)}\ or\ {R}_{(dark)}=\frac{\left(\frac{\left({oxy}_{light\vee dark}\right)}{h}-\frac{\left({blank}_{light\vee dark}\right)}{h}\right)\bullet v}{SA} \end{equation*}

We further added measured respiration (R) rates to net photosynthesis rates (P_net_) using equation 2 to calculate gross photosynthesis (P_gross_), assuming that R is constant during the day.


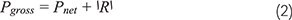


### Endosymbiotic Symbiodiniaceae analysis

To obtain coral tissue slurry for algal symbiont analyses, we defrosted the fragments for 30 min, which had been previously frozen at −20°C. Subsequently, we removed the coral tissue from each fragment using high-pressure air at 5 bar and 10 ml MilliQ® water, using a MasterS68 airbrush.

#### Chlorophyll concentrations by UV-spectrophotometry

To extract chlorophyll *a* from the algal cells, we processed each tissue slurry with a vortex for homogenization and centrifuged (Eppendorf #5424R) the samples at 5000 rpm for 5 min at 4°C before adding 2 ml of 90% Acetone to the pellets. We then incubated all samples for 24 hour at 4°C, after which we added them to a BRANSON 5510-MT Ultrasonic cleaner in an ice water bath for another 10 min. For analysis, we measured the absorbance of all samples in duplicates at two fixed wavelengths, 660 and 663 nm, with a UV-spectrophotometer (Thermo Fisher Scientific; Multiskan SkyHigh; 1550-801435C). For this, we followed the method by [50] and used their pigment-specific absorption coefficients for the determination of chlorophyll *a* and *c_2_* concentration in dinoflagellates, a method previously used for hard coral tissue extracts [51]. All described steps were conducted in a dark room under minimal light conditions.

#### Algal cell density counts by flow cytometry

To count the density of Symbiodiniaceae cells, we vortexed each coral slurry and made pellets with 100 ﻿μl slurry per fragment to separate the symbionts by centrifuging the tubes with an Eppendorf 5424R microcentrifuge at 8000 rpm for 5 min. We then added 1 mL of seawater and vortexed for another 5 min to homogenize the sample. After removing larger cells/debris with a cell strainer of 30 μm mesh size, we pipetted 200 μl onto a microplate in triplicate (200 μl x 3 wells = 600 μl /sample). For the analysis of cell density per sample we used Flow Cytometry. The Flow Cytometer (BD Accuri™; C6; BD CSampler) was set to 1000 events at 2 min, fluidics set as fast and the threshold as fl3-H = 1200. The wash settings of the samples were set as one cycle. We then added 5 μl to every sample as duplicates. Between every six samples, the well plates were shaken. If the events and plot 2 were higher than 1000 we stopped the procedure and unclogged or backflushed.

#### Photosynthetic efficiency (*F*_*V*_*/F*_*m*_) by pulse amplitude modulation fluorometry

To collect *F_V_/F_m_* data, we measured coral fragments using an Imaging pulse amplitude modulation (PAM) (Heinz Walz GmbH; model IMAG-K7; IMAG-MAX/L #LRLB0534B) after a dark adaptation period of 1 h to prevent disturbances of daytime photoinhibition artefacts and to allow for the photo reaction centres to recover thoroughly. The maximum quantum yield of PSII photochemistry was set as the chlorophyll fluorescence parameter *F_V_/F_m_*, a ratio of variable to maximum fluorescence after dark adaptation, using equation 3.


(3) \begin{equation*} Fv= Fm- Fo \end{equation*}

F*_m_* is the maximum fluorescence level that was perceived with a brief saturating pulse of actinic light, while Fo accounted for the initial fluorescence signal that the PAM recognized via measuring light. Per PAM measurement, five *F_V_/F_m_* points were randomly set onto each coral. PAM settings were remained the same throughout all measurements and set as the following: measuring light intensity (MI) = 7, saturation pulse intensity (SI) = 7, saturation pulse width FoFm (SW) = ﻿10, gain (G) = 2, and damping (D) = 2.

### Environmental background data

To ensure continuous monitoring of seawater temperature at the coral probiotics village throughout the experiment, a conductivity, temperature, and depth sensor (Multiparameter CTD, Ocean Seven 310, Idronaut, Italy) was deployed at the research site. The in situ seawater temperature at the probiotics village ranged from 24.8°C to 27.7°C between February and April 2023. The average daily temperature on the days when coral fragments were collected for the experiment ranged from 25.5°C during the first collection (09/02/23 for CT26) to 26.1°C on the last collection day (03/03/23 for BMC32). On all coral fragment collection days (09/02/23 for CT26; 21/02/23 for BMC26; 26/02/23 for CT32; 03/03/23 for BMC32), seawater temperatures showed colder average temperatures compared to the following month where temperatures mostly increased by +1°C.

### Statistical analyses

RStudio (Version 2023.03.0 + 386) was used for statistical analysis. Survival response parameters were assessed using Pearson’s Chi-squared test and pairwise comparisons of proportions among treatments (CT26, BMC26, CT32, BMC32). One-way ANOVA was conducted to analyse treatment effects on primary production, chlorophyll *a* and c_2_ and Symbiodiniaceae density. Linear hypotheses were tested with a 95% confidence level, and post-hoc pairwise comparisons utilized Tukey’s test. Assumption tests included Shapiro-Wilkinson for normal distribution and Levene’s for homogeneity. Logarithmic transformation addressed non-normality in BMC26 Symbiodiniaceae density. Data normality violations led to Kruskal-Wallis tests, with post-hoc Dunn’s tests and Bonferroni-adjusted *P*-values for chlorophyll *a* (*P. verrucosa)* and *F_V_/F_m_* (both species). Water quality parameters were tested using a linear mixed-effects model, accounting for tank variability as a random effect. Data was plotted with the ggplot function in the “ggplot2” package. Significance was set at *P* < 0.05, and results refer to mean ± standard deviation (SD).

## Results

### Water quality parameters

We found no statistically significant variability between salinity and temperature measurements among treatments for the coral tanks of *Acropora* cf. *hemprichii* and *P. verrucosa* (Mixed-effects model, *P* > 0.05).

### Host survival

**Figure 2:**
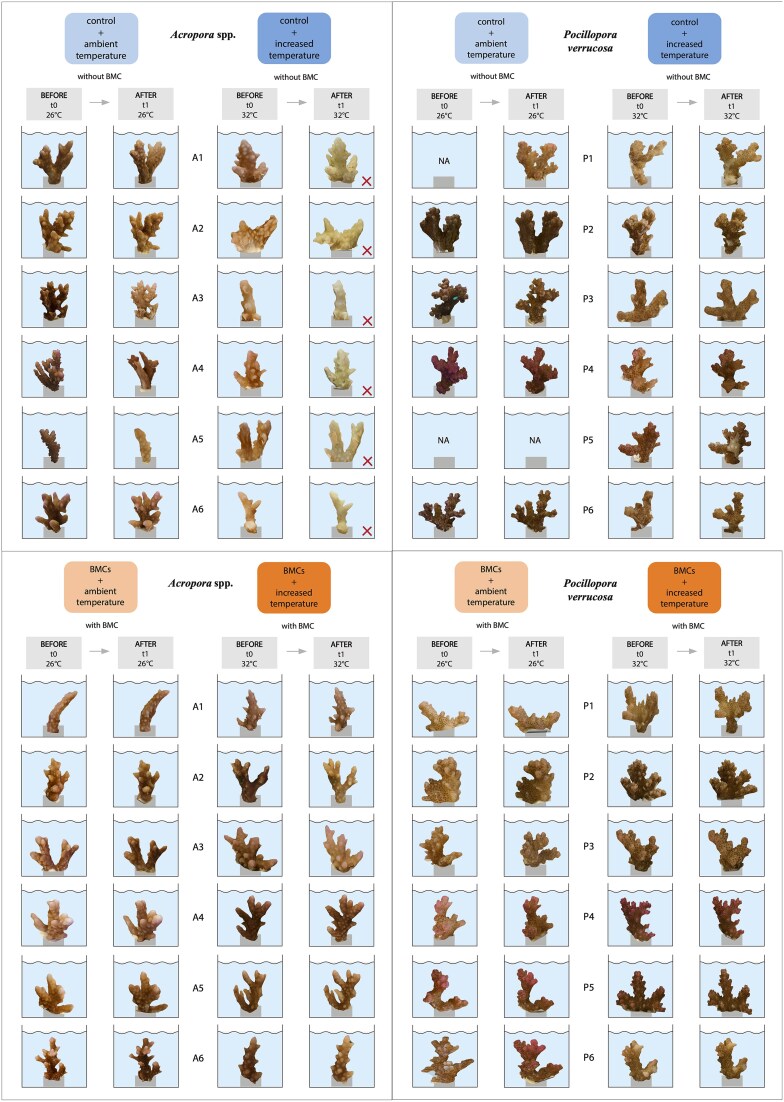
Comparative 3D models showing how BMCs prevent mortality of *Acropora* cf. *hemprichii* fragments (left) compared to *P. verrucosa* fragments (right). Illustrated are the following treatments: CT26 (control at 26°C, light blue), CT32 (control at 32°C, dark blue), BMC26 (BMC addition at 26°C, light orange), BMC32 (BMC addition at 32°C, dark orange). “t0” refers to the commencement of the experiment and “t1” refers to the conclusion of the experiment, precisely 48 h after the commencement. The designations A1–6 and P1–6 represent the specific colonies from which the fragments originated. Fragments labeled with “NA” were unavailable due to complications during the dives. Fragments marked with a red cross were classified as dead.

BMC and control treatments at 26°C exhibited 100% survival throughout the 48-hour experimental period in both coral species. At 32°C, all fragments of *Acropora* cf. *hemprichii* in the control treatment showed white colouration and severe necrosis with >75% tissue loss (Fig. 2, upper, A1–6) with a survival of 0 ± 0% (mean ± SD). In contrast, *Acropora* cf. *hemprichii* fragments inoculated with BMCs had fully intact tissue and 100 ± 0% survival (Fig. 2, lower, P1–6). We did not detect any difference in survival between control and BMC-treated *P. verrucosa* at 32°C, despite minor signs of necrosis on three control fragments (Fig. 2, lower, P1, P2, P4). Analysis revealed a significant association between treatment and survival for *Acropora* cf. *hemprichii* (Pearson Chi-squared test, χ^2^(3) = 24, *P* < 0.001), while there was no variation in *P. verrucosa* survival. The survival of *Acropora* cf. *hemprichii* at 32°C in the control was significantly lower compared to fragments of all other treatments (Pairwise comparisons of proportions test, *P* = 0.0039).

### Primary production

**Figure 3:**
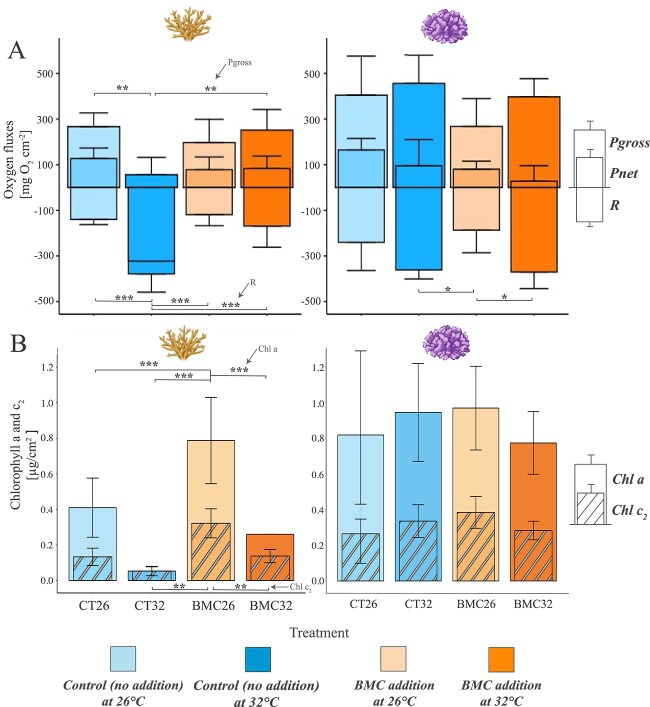
These bar charts visualise the distribution of a) the gross photosynthesis (P_gross_), net photosynthesis (P_net_) and respiration (R), and B) the distribution of the chlorophyll a (banded coloured box) and c_2_ (coloured box) concentrations for Acropora cf. hemprichii (left) and *P. verrucosa* (right) under experimental conditions. Error bars represent standard deviation. ^*^^*^^*^ < 0.001 ^*^^*^ < 0.01 ^*^ < 0.05.

Results from the visual survival response were confirmed by primary production processes. *Acropora* cf. *hemprichii* was strongly affected by the acute stress of 32°C in the control as seen in the lowest P_gross_ values of 56 ± 83 mg O_2_ cm^−2^ h^−1^, being 4–5 times lower than for both treated fragments at the same temperature and control fragments exposed to 26°C, and 3–4 times lower than treated fragments at 26°C. *P. verrucosa* showed a contrasting trend as fragments were doing exceptionally well in terms of P_gross_ with the highest values for control fragments at 32°C of 456 ± 124 mg O_2_ cm^−2^ h^−1^. P_gross_ showed significant differences across treatments for *Acropora* cf. *hemprichii* (one-way ANOVA, F [2, 15] = 7.39, *P* < 0.01), but not for *P. verrucosa* (*P* > 0.05)*.* While we did not observe differences between control and BMC-inoculated fragments at 26°C (*P* > 0.05), it is noteworthy that BMC-inoculated *Acropora* cf. *hemprichii* at 32°C had significantly higher mean values for P_gross_ compared to the control (Post-hoc Tukey test, *P* ≤ 0.05) (Fig. 3, A, left).

The opposite trend was observed for R, as *Acropora* cf. *hemprichii* at 32°C in the controls showed the highest R, being 2–3 times higher than the other *Acropora* cf. *hemprichii* treatments. We detected minimal variation in R for *P. verrucosa* treatments, with the treated fragments at 32°C exhibiting slightly higher R rates compared to the other treatments, albeit only by a factor of 1–2. BMC treatment had a significant effect on R for both *Acropora* cf. *hemprichii* (F [3, 16] = 13.456, *P* < 0.001) (Fig. 3, A, left) and *P. verrucosa* (One-way ANOVA, F [3, 19] = 5.216, *P* = 0.00851) (Fig. 3, A, right). The mean R values of the 26°C treatments showed no significant differences for both species (*P* > 0.05). At 32°C, treated *Acropora* cf. *hemprichii* demonstrated significantly higher respiration rates compared to their counterparts in the controls (Post-hoc Tukey test, *P* < 0.001). *P. verrucosa* displayed notably higher respiration rates in treated fragments at 32°C compared to treated fragments at 26°C (Post-hoc Tukey test, *P* = 0.018), as well as in control fragments at 32°C compared to treated fragments at 26°C (Post-hoc Tukey test, *P* = 0.025). In summary, these findings highlight significant variations in primary production amongst treatments, specifically in gross photosynthesis and respiration for *Acropora* cf. *hemprichii,* and solely in respiration for *P. verrucosa*.

### Chlorophyll *a* and c_2_ concentrations

We detected the lowest chlorophyll *a* concentration for *Acropora* cf. *hemprichii* exposed to 32°C in the controls with 0.05 ± 0.02 μg/cm^2^, being 4–5 times lower than treated fragments at 32°C, 7–8 times lower than control fragments at 26°C and 14–15 times lower than treated fragments at 26°C. Regarding chlorophyll c_2_, *Acropora* cf. *hemprichii* subjected to the 32°C control treatment exhibited the lowest concentration at 0.05 ± 0.02 μg/cm^2^, which was 2 to 3 times lower in control fragments at 26°C and treated fragments at 32°C as well as 6 times lower in treated fragments at 26°C. Subsequently, there were significant differences across *Acropora* cf. *hemprichii* treatments for chlorophyll *a* (Kruskal-Wallis chi-squared, χ^2^ = 18.829, df = 3, *P* < 0.001) and c_2_ concentrations (One-way ANOVA, F [3, 20] = 22.94, *P* < 0.001). Although concentrations of *Acropora* cf. *hemprichii* control fragments compared to treated fragments at 32°C were lower, there was no statistically significant distinction observed for both chlorophyll *a* (Dunn’s test, *P* = 1) and chlorophyll c_2_ (Post-hoc Tukey test, *P* = 1). Nonetheless, the influence of the increased temperature treatment on the concentration of chlorophyll *a* in treated *Acropora* cf. *hemprichii* was evident, with significantly lower values observed at 32°C compared to treated corals at 26°C (Dunn’s test, *P* = 0.002). Despite exposure to acute thermal stress, treated *Acropora* cf. *hemprichii* corals at 32°C demonstrated almost identical chlorophyll c_2_ values to the control group at ambient temperature, measuring 0.138 ± 0.03 and 0.133 ± 0.04 μg/cm^2^, respectively. Regarding chlorophyll c_2_, the concentration of treated *Acropora* cf. *hemprichii* corals at 26°C exhibited an exceptional level of 0.322 ± 0.08 μg/cm^2^, which was significantly higher compared to all other treatments (Post-hoc Tukey test, *P* < 0.001) (Fig. 3, B, left). There were no statistical differences between *P. verrucosa* treatments for both chlorophyll *a* (One-way ANOVA, *P* > 0.05) and c_2_ (One-way ANOVA, *P* > 0.05) (Fig. 3, B, right).

### Symbiodiniaceae cell density

**Figure 4:**
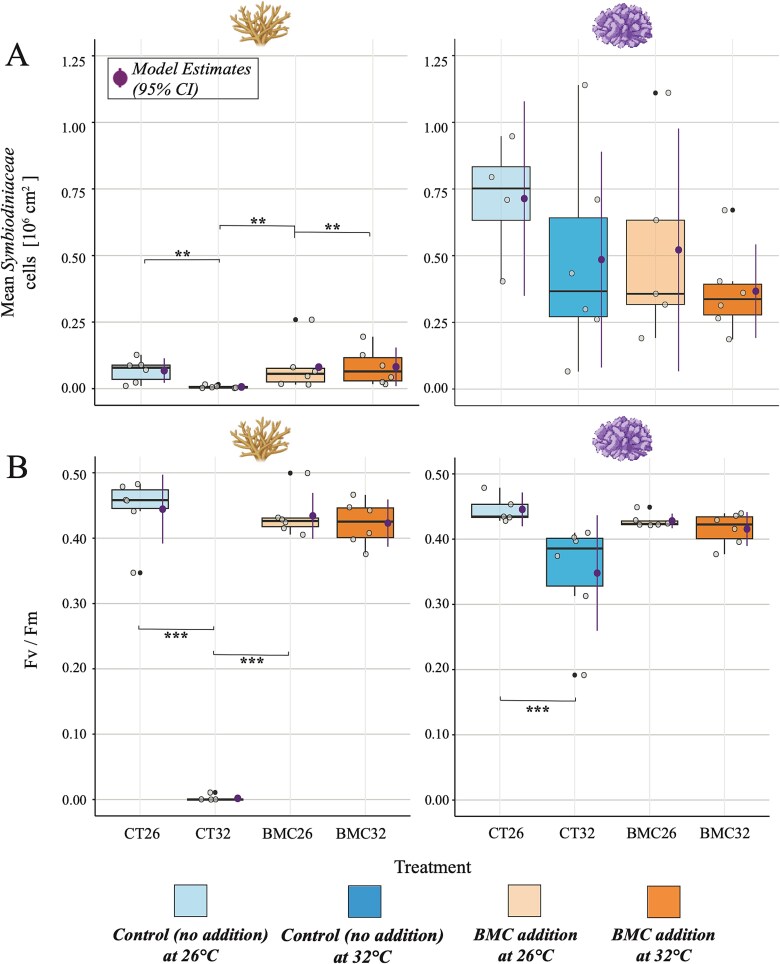
A) Mean Symbiodiniaceae cell density, and B) the maximum PSII quantum yield (F_V_/F_m_) for *Acropora* cf. *hemprichii* (left) and *Pocillopora verrucosa* (right) under experimental conditions. Each boxplot shows the interquartile range (coloured box), the median (black central line), the lower and upper extremes (black whiskers), the raw data (grey dots), and model estimates with a 95% confidence interval (purple dot and line). Outliers, if present, are shown as black dots (>1.5 inter-quartile range from third quartile). ^*^^*^^*^ < 0.001 ^*^^*^ < 0.01 ^*^ < 0.05.

The cell density data of Symbiodiniaceae also correlates with our survival results and confirms the observed trend in colouration among the different treatments. Notably, *Acropora* cf. *hemprichii* fragments at 32°C in the controls, previously categorized as “dead”, exhibited a significant reduction or complete absence of Symbiodiniaceae within the holobiont after 48 h of acute heat stress. This is contrasting to all other *Acropora* cf. *hemprichii* treatments, where Symbiodiniaceae cells were extremely dense, being 10–11 times higher for control fragments at 26°C, and 12–13 times higher for treated fragments at 26°C and 32°C when compared to control fragments at 32°C. Between the two coral species, the overall cell density was higher for *P. verrucosa* with 0.831 ± 0.293 x 10^6^ cm^−2^ (control fragments at ambient temperature) being 12 times higher than the same treatment of *Acropora* cf. *hemprichii.* Following all other measured response parameters, *P. verrucosa* was not affected by acute heat stress and thus Symbiodiniaceae remained relatively stable across treatments (Fig. 4, A, right). We found a significant difference for Symbiodiniaceae cell density across treatments for *Acropora* cf. *hemprichii* (One-way ANOVA, F [3, 20] = 9.437, *P* < 0.001), but not for *P. verrucosa* (One-way ANOVA, *P* = 0.147). *Acropora* cf. *hemprichii* fragments at 32°C in the control treatment exhibited significantly lower mean Symbiodiniaceae cell densities compared to control (Post-hoc Tukey test, *P* ≤ 0.01) and BMC-inoculated fragments at 26°C (Post-hoc Tukey test, *P* ≤ 0.01), while treated fragments at 32°C were significantly higher than treated fragments at 26°C (Post-hoc Tukey test, *P* ≤ 0.01) (Fig. 4, A, left).

### Photosynthetic efficiency

We detected the highest *F_V_/F_m_* values for control fragments at 26°C. Both *Acropora* cf. *hemprichii* and *P. verrucosa* demonstrated comparable efficiencies, with *F_V_/F_m_* values of 0.44 ± 0.04 and 0.44 ± 0.01, respectively. With a temperature increase of +6°C (from 26°C to 32°C), *F_V_/F_m_* exhibited substantially lower values, dropping by 99% for the control fragments of *Acropora* cf. *hemprichii* and by 28% for the controls of *P. verrucosa*. The addition of BMCs to heat-stressed fragments at 32°C resulted in the maintenance of *F_V_/F_m_* for both coral species, with values approximating those observed under ambient conditions. We found a significant difference of *F_V_/F_m_* across treatments for both *Acropora* cf. *hemprichii* (Kruskal-Wallis chi-squared, χ^2^ = 14.089, df = 3, *P* = 0.002) and *P. verrucosa* (Kruskal-Wallis chi-squared, χ^2^ = 12.729, df = 3, *P* = 0.005). *F_V_/F_m_* of *Acropora* cf. *hemprichii* control fragments at 32°C were statistically lower than for control fragments at 26°C (Dunn’s test, *P* < 0.001) and BMC-inoculated fragments at 26°C (Dunn’s test, *P* < 0.001) (Fig. 4, B, left). For *P. verrucosa* control fragments at 32°C were only significantly lower than their counterparts in the control treatment at 26°C (Dunn’s test, *P* < 0.001) (Fig. 4, B, right). A summarized overview of the results of all response parameters can be found in Table 1, with the full dataset available in Supplement 1.

**Table 1:** Summarized overview of all results (mean ± SD) assigned to the respective analysis.

Analysis	*Coral holobiont analyses*			*Endosymbiotic Symbiodiniaceae* analysis			
Response parameter	**Survival (%)**	**Primary production**		**Chlorophyll**		***Symbiodinia- ceae* cell density (10** ^**6**^ **cm**^**−2**^**)**	**Photosynthetic efficiency (*F***_***V***_ ***/F***_***m***_ **)**
		**Gross photosynthesis (h cm** ^**2**^**)**	**Respiration (h cm** ^**2**^**)**	**Chl *a* (μg/cm** ^**2**^**)**	**Chl c**_**2**_ **(μg/cm**^**2**^**)**		
*Acropora* cf. *hemprichii*							
**Control + 26°C**	100 ± 0	266.87 ± 60.09	−139 ± 22	0.40 ± 0.16	0.13 ± 0.04	0.06 ± 0.04	0.44 ± 0.04
**Control + 32°C**	0 ± 0	56.18 ± 75.95	−378.48 ± 79.93	0.05 ± 0.02	0.05 ± 0.04	0.00 ± 0.00	0.00 ± 0.00
**BMCs + 26°C**	100 ± 0	196.88 ± 102.17	−118.88 ± 48.54	0.78 ± 0.24	0.32 ± 0.07	0.08 ± 0.08	0.43 ± 0.03
**BMCs + 32°C**	**↑ 100 ± 0**	**↑ 251.63 ± 90.42**	**↓ -168.70 ± 93.05**	0.26 ± 0.00	0.13 ± 0.03	**↑ 0.08 ± 0.06**	0.42 ± 0.03
*Pocillopora verrucosa*							
**Control + 26 °C**	100 ± 0	404.62 ± 171.10	−239 ± 123	0.81 ± 0.47	0.26 ± 0.14	0.83 ± 0.29	0.44 ± 0.01
**Control + 32°C**	100 ± 0	456.12 ± 124.79	−360.94 ± 39.42	0.94 ± 0.27	0.33 ± 0.08	0.48 ± 0.35	0.34 ± 0.07
**BMCs + 26°C**	100 ± 0	268.09 ± 122.03	−186.75 ± 99.24	0.96 ± 0.23	0.38 ± 0.07	0.52 ± 0.32	0.42 ± 0.00
**BMCs + 32°C**	100 ± 0	397.60 ± 79.57	−370.22 ± 72.55	0.77 ± 0.17	0.28 ± 0.04	0.36 ± 0.15	0.41 ± 0.02

Numbers in bold show significant increase (↑) or decrease (↓) for holobiont function of “BMC + 32 °C” (BMC addition at increased temperature) compared to “control + 32 °C” (no addition at increased temperature).

## Discussion

Probiotics, i.e. BMCs, could aid ongoing coral conservation and restoration efforts facing the coral reef crisis by reducing coral bleaching and mortality under conditions of thermal stress [26]. The present study reveals that BMCs exhibit positive effects during short-term acute heat stress lasting only 2 days, building on previous findings that explored BMCs effect during mid-term heat stress lasting 26, 34, and 39 days [30, 32, 34] as well as during long-term heat stress lasting 75 days [35]. Here, we successfully demonstrate the ability of BMCs to help prevent mortality, maintain primary production processes in proximity to non-stress conditions, and positively influence the photo-physiology of *Acropora* cf. *hemprichii* during an acute heat-stress experiment. Contrastingly, the coral holobiont of *P. verrucosa* showed no signs of heat stress during the experiments and, therefore, no discernible effect of BMC treatment.

### Rapid response of Acropora cf. *hemprichii* to BMCs inoculation during heat stress exposure resulted in reduced mortality

*Acropora* cf. *hemprichii* inoculated with BMCs at 32°C exhibited a remarkable 100% survival after two days, presenting a substantial increase compared to their conspecifics in the control treatments. Our findings are consistent with those of [35], who showed a 40% rise in coral survival for *Mussismilia hispida* at 30°C over 75 days with BMC inoculation. However, the difference in decreased mortality observed between the studies could be attributed to variances in coral species and BMC consortiums, and variations in the intensity, duration, and nature of heat exposure.

The rapid response of *Acropora* cf. *hemprichii* in incorporating BMCs within 2 days was not surprising as the coral host’s capacity to acquire microorganisms from the surrounding seawater is enhanced under stress conditions, as indicated by a decreased organization of the microbiome [42, 52]. During a reciprocal transplantation experiment followed by short-term heat exposure, [53] observed rapid shifts in the composition of the bacterial community of *Acropora hyacinthus*, a phenomenon evident after 20 hours, indicating that bacterial community dynamics can be significantly influenced by acute short-term heat exposure. Fragment collection in the current study further coincided with the coldest time of the year, marked by a recorded low temperature at 24.8°C at the research site. We hypothesize that potential acclimatization to winter temperatures [54] likely impacted coral sensitivity to heat stress and contributed to the high mortality in the controls at 32°C. The pre-heat exposure temperature conditions underscore the significance of considering various stress paradigms and their potential influence on the observed outcomes of probiotic treatments on corals.

### BMCs have positive effects on primary production and photo-physiology of severely heat-stressed *Acropora* cf. *hemprichii*

BMC inoculation before heat exposure revealed comparable rates of gross photosynthesis and respiration in *Acropora* cf. *hemprichii* compared to their conspecifics under non-stress conditions. These rates were consistently 4–5 times higher and 2–3 times lower, respectively, when compared to the control treatment at 32°C. The observed trend is confirmed by the collective outcomes of photo-physiological responses, which indicate BMCs ability to exert a positive effect on Symbiodiniaceae in *Acropora* cf. *hemprichii* under acute heat stress conditions, thereby maintaining coral holobiont function. This beneficial effect was evident in corals inoculated with BMCs at 32°C, in contrast to the control, which displayed impaired holobiont function, as indicated by reduced chlorophyll *a* and c_2_ concentrations, Symbiodiniaceae cell densities and photosynthetic efficiency. The observed stabilizing effect of BMCs on the photosynthetic performance of Symbiodiniceae in our study aligns with findings in [30, 32, 34, 35], who utilized F_V_/F_m_ ratios as a coral health indicator through diving PAM. [34] also noted BMC-induced improvements in Symbiodiniaceae cell density, while [30] detected significant enhancements in primary production.

No comparable study has yet investigated the effect of BMCs on chlorophyll *a* and c_2_ concentrations. [55] reported chlorophyll *a* concentration for *A. hemprichii* at 27°C as 0.76 ± 0.09 μg cm^−2^, significantly higher than our control group findings at 26°C (0.40 ± 0.16 μg cm^−2^). However, these concentrations closely resemble those observed in BMC-inoculated *Acropora* cf. *hemprichii* at the same temperature (0.78 ± 0.24 μg cm^−2^). Symbiodiniaceae cell density was markedly lower than observed in previous data recovered from *A. hemprichii* [56] despite trend coherence across other response parameters. This discrepancy may be attributed to incomplete homogenization of coral tissue by the vortex.

The observed positive effect of BMCs on the photo-physiology of *Acropora* cf. *hemprichii* may be attributed to the ability of beneficial microorganisms to restore homeostasis and ensure an internal equilibrium between Symbiodiniaceae and the coral host [27]. The restoration of homeostasis is crucial for maintaining stable photosynthetic activity [57] and is achieved by BMCs inoculation through (i) the modulation of microbiota composition, (ii) pathogen control, and (iii) the enhancement of nutrient availability, amongst others [26]. Building on evidence that coral holobionts selectively engage with their microbial partners [58] and observations of *A. hemprichii* altering its microbial community in response to acute heat stress within 20 hour [53], we believe that *Acropora* cf. *hemprichii* may have adapted to the new thermal condition through different mechanisms associated with probiotic inoculation [59]. We further hypothesize that (i) microbiome restructuring and (ii) pathogen control may have been achieved through BMC inoculation and resource competition between the beneficial microorganisms present in the probiotic treatment and pathogens. By vying for essential resources such as nutrients and space, beneficial microorganisms effectively limit the availability of these resources to pathogens, thereby suppressing their growth and proliferation [60]. We believe (ii) was further achieved through the bacterial strains capability to directly inhibit pathogens by producing antimicrobial compounds, which is crucial for coral host health and the shaping of microbial community structure [61]. Finally, we propose that BMCs had an important input in 3. enhancing nutrient availability (e.g. nitrogen) through their involvement in nutrient cycling [27] and acquisition [26], thereby contributing significantly to the successful maintenance of primary production processes in heat-stressed *Acropora* cf. *hemprichii.* Bacterial strains within the BMC II consortium, such as *H. piezotolerans* and *Cobetia sp.*, have been found to possess *nifH* gene and *nirK* gene. The presence of these genes, or other potential marker genes such as *nirS,* may enhance the nitrogen cycle as they are associated with nitrogen fixation and denitrification [56, 62]. This could facilitate the preservation of an N-limited state, which is essential for sustaining primary production processes [63]. Further research is necessary to substantiate these hypotheses and define the specific short-term protective mechanisms associated with the N cycle triggered by BMC inoculation.

### *P. verrucosa* exhibits remarkable heat tolerance, leading to no discernible effects of BMC treatment on the coral holobiont function

*P. verrucosa* showed to be significantly heat resistant with high survival, exhibiting 100 ± 0% (mean ± SD) for both BMC-inoculated and control fragments under heat stress, only minor signs of tissue necrosis, fully intact primary production processes and no significant alterations in terms of chlorophyll *a* and c_2_ concentrations, *Symbiodiniceae* cell density, and *F_V_/F_m_* ratios.

As a result, the full potential benefits of a probiotic treatment could not be realized. We suggest additional *ex situ* experiments using different temperature regimes and prolonged exposure times for *P. verrucosa* to unlock the full potential of BMCs under short-term heat stress.

Our result aligns closely with that of [40], who similarly found no effect of BMCs on the coral physiology of *P. verrucosa* as assessed by photosynthetic efficiency of *Symbiodiniceae* and coral thermal tolerance*,* most likely as the in situ study was not affected by bleaching or disease leaving BMC-inoculated coral fully intact. These findings may indicate that BMC I consortium poses no apparent harm on fully intact *P. verrucosa* holobionts considering the health indicators measured in this study, while BMCs could still provide a restructuring of the microbiome [40] and potentially epigenome [64]. The result of unchanged photo-physiology for *P. verrucosa* at 32°C may be explained by acclimatization or adaptation of *Symbiodiniceae,* allowing colonies to cope with short-term exposure to high temperatures. This is based on findings by [65], suggesting that the predominant endosymbiont algal species of *P. verrucosa* in the Red Sea, *Symbiodinium microadriaticum*, has undergone acclimatization or adaptation to the specific environmental conditions found within different regions of the Red Sea. Even though *P. verrucosa* predominantly inhabits the Northern and Central Red Sea, colonies can be found in smaller abundances in the Southern region, where reefs can thrive at temperatures as high as 31°C [65].

While our methodology is entirely replicable, it is important to acknowledge that the coral fragments used in this study were collected from six colonies of a specific offshore reef in the central Red Sea. Therefore, the results obtained may not necessarily apply to other regions due to potential variations in the response of coral holobionts to heat stress [66]. Additional studies expanding on the number of species, areas, and experiments can further validate our results and underlying mechanisms of coral protection. Recognizing the challenge of unknown starting conditions, we emphasize the importance of incorporating baseline assessments in future studies to better standardize initial natural biological variability and improve the reliability of experimental outcomes. Still, the observed possibility of obtaining short-term BMC effects on stressed corals will support and guide additional studies and management strategies aimed at conserving heat-sensitive corals in natural reef ecosystems, emphasizing the potential utility of proactive probiotic interventions.

## Supplementary Material

Supplement_1_Data_ycaf039

## Data Availability

All data generated or analysed during this study are included in this published article and its supplemental material.

## References

[1] Cesar HSJ, Burke L, Pet-Soede L. The Economics of Worldwide Coral Reef Degradation Cesar Environmental Economics Consulting (CEEC), 2003.

[2] Ferrario F, Beck MW, Storlazzi CD et al. The effectiveness of coral reefs for coastal hazard risk reduction and adaptation. 2014;5:3794. 10.1038/ncomms4794 PMC4354160 24825660

[3] Spalding M, Burke L, Wood SA et al. Mapping the global value and distribution of coral reef tourism. 2017;82:104–13. 10.1016/j.marpol.2017.05.014

[4] Burke L, Reytar K, Spalding M et al. Reefs at risk revisited. 2011.

[5] Wiedenmann J, D'Angelo C, Smith EG et al. Nutrient enrichment can increase the susceptibility of reef corals to bleaching. *Nat Clim Chang* 2013;3:160–4. 10.1038/nclimate1661

[6] Riegl B, Purkis S. Coral population dynamics across consecutive mass mortality events. *Glob Chang Biol* 2015;21:3995–4005. 10.1111/gcb.13014 26119322

[7] Morris LA, Voolstra CR, Quigley KM et al. Nutrient availability and metabolism affect the stability of coral–Symbiodiniaceae symbioses. *Trends Microbiol* 2019;27:678–89. 10.1016/j.tim.2019.03.004 30987816

[8] Genin A, Levy L, Sharon G et al. Rapid onsets of warming events trigger mass mortality of coral reef fish. *Proc Natl Acad Sci* 2020;117:25378–85. 10.1073/pnas.2009748117 32958634 PMC7568245

[9] Donovan MK, Burkepile DE, Kratochwill C et al. Local conditions magnify coral loss after marine heatwaves. *Science* 2021;372:977–80. 10.1126/science.abd9464 34045353

[10] Hylkema A, Kitson-Walters K, Kramer PR et al. The 2022 Diadema antillarum die-off event: comparisons with the 1983-1984 mass mortality. *Front Mar Sci* 2023;9:9. 10.3389/fmars.2022.1067449

[11] Heron SF, Maynard JA, van Hooidonk R et al. Warming trends and bleaching stress of the world’s coral reefs 1985–2012. *Sci Rep* 2016;6:38402. 10.1038/srep38402 27922080 PMC5138844

[12] Hughes TP, Kerry JT, Álvarez-Noriega M et al. Global warming and recurrent mass bleaching of corals. *Nature* 2017;543:373–7. 10.1038/nature21707 28300113

[13] Duarte GAS, Villela HDM, Deocleciano M et al. Heat waves are a major threat to turbid coral reefs in Brazil. *Front Mar Science* 2020;7:7. 10.3389/fmars.2020.00179

[14] Gates RD, Baghdasarian G, Muscatine L. Temperature stress causes host cell detachment in symbiotic cnidarians: implications for coral bleaching. *Biol Bull* 1992;182:324–32. 10.2307/1542252 29304594

[15] Hoegh-Guldberg O . Climate change, coral bleaching and the future of the world’s coral reefs. *Mar Freshw Res* 1999;50:839–66. 10.1071/MF99078

[16] Douglas AE . Coral bleaching––how and why? *Mar Pollut Bull* 2003;46:385–92. 10.1016/S0025-326X(03)00037-7 12705909

[17] Muscatine L . The role of symbiotic algae in carbon and energy flux in reef corals. *Ecosyst World* 1990;25:75–87.

[18] IPCC . Climate Change 2023: Synthesis Report. Geneva, Switzerland. 2023. 10.1186/s13750-023-00311-4.

[19] Buddemeier RW, Fautin DG. Coral bleaching as an adaptive mechanism. *Bioscience* 1993;43:320–6. 10.2307/1312064

[20] Rosenberg E, Ben-Haim Y. Microbial diseases of corals and global warming. *Environ Microbiol* 2002;4:318–26. 10.1046/j.1462-2920.2002.00302.x 12071977

[21] Rosenberg E, Falkovitz L. The Vibrio shiloi/Oculina patagonica model system of coral bleaching. *Ann Rev Microbiol* 2004;58:143–59. 10.1146/annurev.micro.58.030603.123610 15487933

[22] Reshef L, Koren O, Loya Y et al. The coral probiotic hypothesis. *Environ Microbiol* 2006;8:2068–73. 10.1111/j.1462-2920.2006.01148.x 17107548

[23] Huggett MJ, Apprill A. Coral microbiome database: integration of sequences reveals high diversity and relatedness of coral-associated microbes. *Environ Microbiol Rep* 2018;11:372–85. 10.1111/1758-2229.12686 30094953 PMC7379671

[24] Mohamed AR, Ochsenkühn MA, Moustafa A et al. The coral microbiome: towards an understanding of the molecular mechanisms of coral–microbiota interactions. *FEMS Microbiol Rev* 2023;47. 10.1093/femsre/fuad005 PMC10045912 36882224

[25] Voolstra CR, Raina J, Doerr M et al. The coral microbiome in sickness, in health and in a changing world. *Nat Rev Microbiol* 2024; 22:460–75. 10.1038/s41579-024-01015-3 38438489

[26] Peixoto RS, Rosado PM, De Assis Leite DC et al. Beneficial microorganisms for corals (BMC): proposed mechanisms for coral health and resilience. *Front Microbiol* 2017;8:8. 10.3389/fmicb.2017.00341 28326066 PMC5339234

[27] Peixoto RS, Sweet M, Villela HDM et al. Coral probiotics: premise, promise. *Prospect Revi Anim Biosci* 2021;9:265–88. 10.1146/annurev-animal-090120-115444 33321044

[28] Rosado M, Cardoso P, Rosado JG et al. Exploring the potential molecular mechanisms of interactions between a probiotic consortium and its coral host. *MSystems* 2023;8. 10.1128/msystems.00921-22 PMC9948713 36688656

[29] Doering T, Tandon K, Putchim L et al. Genomic exploration of coral-associated bacteria: identifying probiotic candidates to increase coral bleaching resilience in *Galaxea fascicularis*. *Microbiome* 11:185. 10.1186/s40168-023-01622-x PMC10439622 37596630

[30] Cardoso P, Hill L, Villela HDM et al. Localization and symbiotic status of probiotics in the coral holobiont. *MSystems* 2024. 10.1128/msystems.00261-24 PMC11097643 38606974

[31] Peixoto RS, Voolstra CR. The baseline is already shifted: marine microbiome restoration and rehabilitation as essential tools to mitigate ecosystem decline. *Front Mar Science* 2023;10:10. 10.3389/fmars.2023.1218531

[32] Rosado PM, De Assis Leite DC, Duarte GAL et al. Marine probiotics: increasing coral resistance to bleaching through microbiome manipulation. *ISME J* 2019;13:921–36. 10.1038/s41396-018-0323-6 30518818 PMC6461899

[33] Peixoto RS, Voolstra CR, Sweet M et al. Harnessing the microbiome to prevent global biodiversity loss. *Nat Microbiol* 2022;7:1726–35. 10.1038/s41564-022-01173-1 35864220

[34] Li J, Zou Y, Li Q et al. A coral-associated actinobacterium mitigates coral bleaching under heat stress. *Environ Microbiome* 2023;18:83. 10.1186/s40793-023-00540-7 PMC10668361 37996910

[35] Santoro EP, Borges R, Espinoza JL et al. Coral microbiome manipulation elicits metabolic and genetic restructuring to mitigate heat stress and evade mortality. *Sci Adv* 2021;7. 10.1126/sciadv.abg3088 PMC8363143 34389536

[36] Ushijima B, Gunasekera P, Meyer J et al. Chemical and genomic characterization of a potential probiotic treatment for stony coral tissue loss disease. *Commun Biol* 2023;6. 10.1038/s42003-023-04590-y PMC10079959 37024599

[37] Moradi M, Magalhaes PR, Peixoto RS et al. Probiotics mitigate thermal stress- and pathogen-driven impacts on coral skeleton. *Front Mar Sci* 2023;10:10. 10.3389/fmars.2023.1212690

[38] Zhang Y, Yang Q, Ling J et al. Shifting the microbiome of a coral holobiont and improving host physiology by inoculation with a potentially beneficial bacterial consortium. *BMC Microbiol* 2021;21:323. 10.1186/s12866-021-02385-x PMC8082877 33910503

[39] Doering T, Wall M, Putchim L et al. Towards enhancing coral heat tolerance: a “microbiome transplantation” treatment using inoculations of homogenized coral tissues. *Microbiome* 2021;9:102. 10.1186/s40168-021-01053-6 PMC8103578 33957989

[40] Delgadillo-Ordoñez N, Garcías-Bonet N, Raimundo I et al. Probiotics reshape the coral microbiome in situ without detectable off-target effects in the surrounding environment. *Commun Biol* 2024;7:437. 10.1038/s42003-024-06135-3 PMC11004148 38594357

[41] Garcias-Bonet N, Villela H, García F et al. The coral probiotics village: an underwater laboratory to tackle the coral reefs crisis. *Authorea* 2024. 10.1002/ece3.71558 PMC12231234 40625325

[42] Ziegler M, Grupstra CGB, Barreto MM et al. Coral bacterial community structure responds to environmental change in a host-specific manner. *Nat Commun* 2019;10:3092. 10.1038/s41467-019-10969-5 PMC6626051 31300639

[43] Veron J, Stafford-Smith M. Corals of the World. Townsville: Australian Institute of Marine Science, 2000.

[44] Richards ZT, Dellisanti W, Nuñez Lendo CI. Acropora hemprichii. The IUCN Red List of Threatened Species 2024:e.T132981A165659729.

[45] Nuñez Lendo CI, Luzon K, Johnson et al. Pocillopora verrucosa. The IUCN Red List of Threatened Species 2024:e.T254422741A165766340.

[46] Roik A, Röthig T, Pogoreutz C et al. Coral reef carbonate budgets and ecological drivers in the central Red Sea – a naturally high temperature and high total alkalinity environment. *Biogeosciences* 2018;15:6277–96. 10.5194/bg-15-6277-2018

[47] Voolstra CR, Valenzuela JJ, Turkarslan S et al. Contrasting heat stress response patterns of coral holobionts across the Red Sea suggest distinct mechanisms of thermal tolerance. *Mol Ecol* 2021;30:4466–80. 10.1111/mec.16064 34342082

[48] Casey JM, Connolly SR, Ainsworth TD. Coral transplantation triggers shift in microbiome and promotion of coral disease associated potential pathogens. *Sci Rep* 2015;5:11903. 10.1038/srep11903 PMC4491727 26144865

[49] Bednarz VN, Naumann MS, Niggl W et al. Inorganic nutrient availability affects organic matter fluxes and metabolic activity in the soft coral genus Xenia. *J Exp Biol* 2012;215:3672–9. 10.1242/jeb.072884 22811248

[50] Jeffrey SW, Humphrey JF. New spectrophotometric equations for determining chlorophylls a, b, c_1_ and c_2_ in higher plants, algae and natural phytoplankton. Biochemie Und Physiologie Der Pflanzen 1975;167:191–4.

[51] Ulstrup KE, Berkelmans R, Ralph PJ et al. Variation in bleaching sensitivity of two coral species across a latitudinal gradient on the great barrier reef: the role of zooxanthellae. *Mar Ecol Prog Ser* 2006;314:135–48. 10.3354/meps314135

[52] Sweet M, Ramsey AD, Bulling MT. Designer reefs and coral probiotics: great concepts but are they good practice? *Biodiversity* 2017;18:19–22. 10.1080/14888386.2017.1307786

[53] Ziegler M, Seneca FO, Yum LK et al. Bacterial community dynamics are linked to patterns of coral heat tolerance. *Nat Commun* 2017;8:14213. 10.1038/ncomms14213 PMC5309854 28186132

[54] Scheufen T, Krämer WE, Iglesias-Prieto R et al. Seasonal variation modulates coral sensibility to heat-stress and explains annual changes in coral productivity. *Sci Rep* 2017;7:4937. 10.1038/s41598-017-04927-8 PMC5504023 28694432

[55] Alabyadh A . Effects of Zinc and Vitamin Supplementation on the Coral *Acropora hemprichii* Health and Growth. Master's thesis KAUST Research Repository 2023. http://hdl.handle.net/10754/693228

[56] Tilstra A, El-Khaled YC, Roth F et al. Denitrification aligns with N_2_ fixation in red sea corals. *Sci Rep* 2019;9:19460. 10.1038/s41598-019-55408-z PMC6923481 31857601

[57] Rodriguez-Lanetty M, Wood-Charlson EM, Hollingsworth LL et al. Temporal and spatial infection dynamics indicate recognition events in the early hours of a dinoflagellate/coral symbiosis. *Mar Biol* 2006;149:713–9. 10.1007/s00227-006-0272-x

[58] Bourne DG, Iida Y, Uthicke S et al. Changes in coral-associated microbial communities during a bleaching event. *ISME J* 2008;2:350–63. 10.1038/ismej.2007.112 18059490

[59] Garcias-Bonet N, Roik A, Tierney B et al. Horizon scanning the application of probiotics for wildlife. *Trends Microbiol* 2023;32:252–69. 10.1016/j.tim.2023.08.012 37758552

[60] Krediet CJ, Ritchie KB, Alagely A et al. Members of native coral microbiota inhibit glycosidases and thwart colonization of coral mucus by an opportunistic pathogen. *ISME J* 2013;7:980–90. 10.1038/ismej.2012.164 23254513 PMC3635231

[61] Kvennefors ECE, Sampayo EM, Kerr CA et al. Regulation of bacterial communities through antimicrobial activity by the coral holobiont. *Microb Ecol* 2012;63:605–18. 10.1007/s00248-011-9946-0 21984347

[62] Pogoreutz C, Rädecker N, Cárdenas A et al. Sugar enrichment provides evidence for a role of nitrogen fixation in coral bleaching. *Glob Chang Biol* 2017;23:3. 10.1111/gcb.13695 28429531

[63] Wang J, Douglas AE. Essential amino acid synthesis and nitrogen recycling in an alga-invertebrate symbiosis. *Mar Biol* 1999;135:219–22. 10.1007/s002270050619

[64] Barno AR, Villela HDM, Aranda M et al. Host under epigenetic control: a novel perspective on the interaction between microorganisms and corals. *BioEssays* 2021;43. 10.1002/bies.202170086 34463364

[65] Sawall Y, Kürten B, Hoang BX. Coral communities, in contrast to fish communities, maintain a high assembly similarity along the large latitudinal gradient along the Saudi Red Sea coast. *J Ecosyst Ecogr* 2014;s4:003. 10.4172/2157-7625.S4-003

[66] Voolstra CR, Suggett DJ, Peixoto RS et al. Extending the natural adaptive capacity of coral holobionts. *Nat Rev Earth Environ* 2021;2:747–62. 10.1038/s43017-021-00214-3

